# Mutational analysis of the Notch2 negative regulatory region identifies key structural elements for mechanical stability

**DOI:** 10.1016/j.fob.2015.07.006

**Published:** 2015-07-30

**Authors:** Natalie L. Stephenson, Johanna M. Avis

**Affiliations:** Faculty of Life Sciences, Manchester Institute of Biotechnology, University of Manchester, 131 Princess Street, Manchester M1 7DN, United Kingdom

**Keywords:** NRR, Negative regulatory region, TACE, TNF-alpha-converting enzyme, hN1, human Notch-1 receptor, hN2, human Notch-2 receptor, HD, heterodimerization (HD) domain, LNR, Lin12-Notch repeats, Notch, Negative regulatory region, Heterodimerization domain, HD domain, Atomic force microscopy, Molecular dynamics simulations

## Abstract

The Notch signalling pathway is fundamental to cell differentiation in developing and self-renewing tissues. Notch is activated upon ligand-induced conformational change of the Notch negative regulatory region (NRR), unmasking a key proteolytic site (S2) and facilitating downstream events. The favoured model requires endocytosis of a tightly bound ligand to transmit force to the NRR region, sufficient to cause a structural change that exposes the S2 site. We have previously shown, using atomic force microscopy and molecular dynamics simulations, that application of force to the N-terminus of the Notch2 NRR facilitates metalloprotease cleavage at an early stage in the unfolding process. Here, mutations are made within the heterodimerization (HD) domain of the NRR that are known to cause constitutive activation of Notch1 whilst having no effect on the chemical stability of Notch2. Comparison of the mechanical stability and simulated forced unfolding of recombinant Notch2 NRR proteins demonstrates a reduced stability following mutation and identifies two critical structural elements of the NRR in its response to force – the linker region between Lin12-Notch repeats LNRA and LNRB and the α3 helix within the HD domain – both of which mask the S2 cleavage site prior to Notch activation. In two mutated proteins, the LNRC:HD domain interaction is also reduced in stability. The observed changes to mechanical stability following these HD domain mutations highlight key regions of the Notch2 NRR that are important for mechanical, but not chemical, stability. This research could also help determine the fundamental differences in the NRRs of Notch1 and Notch2.

## Highlights

• Mutations within heterodimerization (HD) domain of Notch2 reduce mechanical stability.
• Structural changes are observed within the LNRA:B linker region/LNRB and αC helix.
• The LNRC:HD domain interaction is also reduced in stability.
• Changes in mechanical stability versus chemical stability are highlighted.

## 1 Introduction

The highly conserved Notch signalling pathway [1,2] enables direct cell–cell communication and instruction of the differentiation pathways of those cells bearing the Notch receptor. Since Notch plays a fundamental role in cell differentiation of both developing and self-renewing tissues, it is not surprising that the signalling pathway is widely implicated in disease, particularly cancers [3–8]. Although it is a relatively simple and direct pathway, it is subject to intricate control mechanisms throughout [9]. Such control mechanisms are less well understood and clearly an important area for investigation.

The canonical Notch signalling pathway relies on a Notch receptor proteolytic cleavage cascade that leads to release of the Notch intracellular domain from the membrane. Once released, the intracellular domain translocates to the nucleus where it can upregulate its target genes. The proteolytic cleavage cascade is initiated when the Notch transmembrane receptor binds to a ligand (Delta/Serrate/Lag-2) [10,11] presented on the surface of a neighbouring cell. Since there is evidence that soluble ligands cannot stimulate the canonical Notch pathway in mammals and flies [12–14], it has been proposed that there is functional significance to the membrane bound localisation of the ligand and its subsequent endocytosis. Indeed, a favoured model for Notch activation is that Notch experiences a mechanical force due to the transendocytosis of the Notch extracellular domain (or ectodomain) into the neighbouring ligand-bearing cell [15–20]. This force is thought to cause a conformational change within a region distal from the ligand interaction site, the juxtamembrane negative regulatory region (NRR) [3]. Crucially, the NRR converts from a resting, autoinhibited conformation [21,22] to one in which metalloproteases can access and cleave at a key regulatory proteolytic site; the S2 site. Following metalloprotease dependent ectodomain shedding [23,24], the γ-secretase complex cleaves the Notch receptor at the intramembrane S3 cleavage site (and additional sites) [25,26], releasing the intracellular domain to participate in downstream signalling events. In humans, there are four Notch receptors, Notch1–Notch4 (hN1–hN4). All four receptors are proposed to share a requirement for the S2 cleavage event for signal activation, induced upon ligand binding.

Recently, we provided proof of feasibility for the mechanical force model of Notch activation by forcibly unfolding single molecules of the hN2 negative regulatory region. On stretching the hN2 NRR in an atomic force microscope (AFM), cleavage at S2 by the metalloprotease TACE can be directly observed *in vitro* [18]. The Notch NRR comprises three Lin12-Notch repeats (LNRs) wrapped around the heterodimerization (HD) domain, which houses the S2 site. The S2 site is consequently buried in this autoinhibited resting conformation. By combining experimental (AFM) data with molecular dynamics simulations, we were able to show that the S2 site will be accessible to metalloprotease fairly early in the unfolding pathway after force application. Simulations within our study, suggested the minimum required structural change that allowed access to the S2 site was the ‘unplugging’ of the linker between the first two LNR modules (LNRA and LNRB) from a position over the S2 site in the HD domain. However, deletion of the LNRA and the LNRA:B linker region was not sufficient to allow cleavage *in vitro*; removal of the LNRB was also required for S2 cleavage within these experiments [21].

The heterodimerization domain is so-called due to an initial cleavage event carried out by a furin-like convertase at an S1 site located in an exposed loop of the domain. This S1 cleavage occurs during processing and transport of the Notch receptor to the cell membrane and, whilst it does not disrupt the core structure of the HD domain (or play any role in receptor signal activation), the domain is effectively divided into two non-covalently associated N- and C-terminal subunits, HD-N and HD-C. The stable core structure of the HD domain comprises a kinked α1-helix and the β4-strand [27], structural elements that actually derive from the different HD-N and HD-C subunits. Mutations that lie within these structural elements or nearby, known for their activating status within the hN1 associated disease T-cell acute lymphoblastic leukemia (T-ALL) [6], have been shown to increase heterodimer dissociation (relative to wild-type) in native or mildly denaturing conditions [28]. Interestingly, in the context of hN2, these mutated constructs failed to increase heterodimer dissociation upon EDTA treatment [40]. This highlights crucial differences between the hN1 and hN2 receptors. However, whilst these differences in chemical stability of the mutations within hN1 and hN2 are clear, little is known about their effect on mechanical stability, which is key if the mechanotransduction hypothesis is true.

**Fig. 1:**
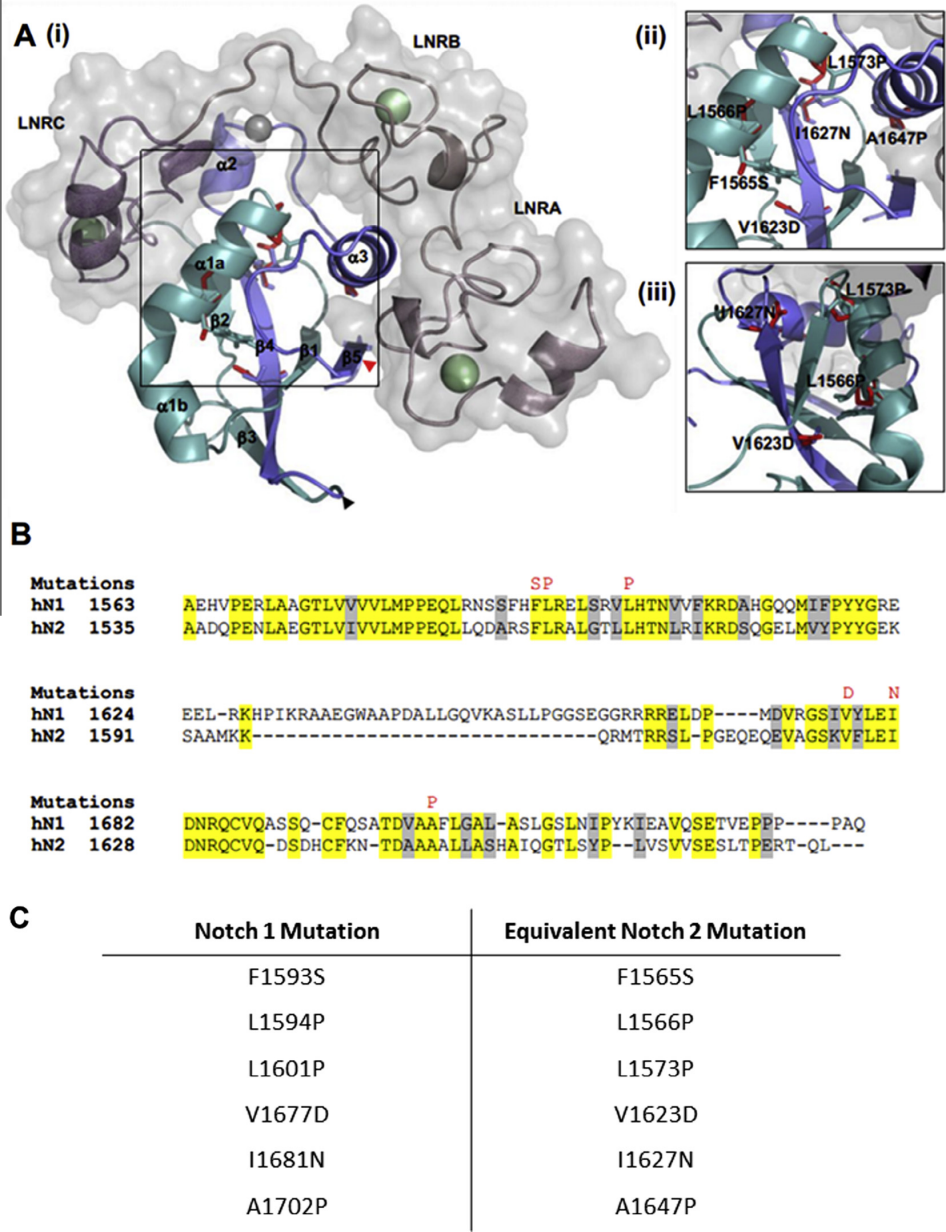
Structure and sequence of the hN2 NRR. (A) (i) hN2-NRR comprising three LNR repeats (cartoon and surface representation) and the HD domain (cartoon representation). S1 cleavage site, which separates the HD domain into the HD-N (turquoise) and HD-C (blue) is shown as a black arrowhead. S2 cleavage site which activates the signalling pathway, is highlighted by a red arrowhead. Black box highlights region enlarged to show mutation sites in (ii) and rotated 90° left in (iii). Wild type residues shown as sticks in domain colours, variant residue shown as sticks in red. (B) Alignment of hN1 and hN2 HD domain sequences, highlighting mutations (red) in residues that are identical (yellow). Conserved residues shown as grey and not conserved residues are shown as white. Diagram created using PyMol 1.3, sequence alignment performed using ClustalW. (C) Table detailing the hN2 mutations analysed within this study, alongside the equivalent mutation found in hN1 T-ALL samples.

Given the increasing evidence for a role for mechanical force in the Notch signal activation mechanism, we investigate the effect of six mutations, related to those shown previously to destabilise the HD domain within hN1, [6,39] on the mechanical stability of recombinant hN2-NRR (hN2 residues 1425–1672). Five of the six mutations are within the core region of the HD domain (Fig. 1A; made up of the β4 strand and α1 helix) which has previously been shown to be a critical region for HD domain stability [27] and at the interface between the HD-N and HD-C. Since our model is the hN2 protein, mutations were chosen based on the sequence homology between hN1 and hN2, ensuring mutation at conserved positions (Fig. 1B). The mutated recombinant hN2-NRR proteins generated are hence: F1565S, L1566P, L1573P, V1623D, I1627N and A1647P (equivalent to the F1593S, L1594P, L1601P, V1677D, I1681N and A1702P in hN1; Fig. 1C). Three of these mutations (L1566P, V1623D and I1627N) have recently been biochemically analysed, revealing fundamental differences between the N1 and N2 NRR [40]. Interestingly, contrary to observations in hN1, none of these three mutations increased HD domain dissociation following EDTA treatment [40], therefore altering ligand independent signalling within hN1 but not in hN2. Here, these mutations will be analysed within hN2 to determine the effect they have on forced unfolding, and therefore potentially ligand induced activation. To do this, we subjected these recombinant hN2-NRR protein constructs with destabilising HD domain mutations to forced unfolding, both in an atomic force microscope and in molecular dynamics (MD) simulations, in order to examine their mechanical properties relative to the wild-type (WT) NRR. The results identify key contributing regions within the HD domain for governing mechanical stability, which could suggest these mutations still affect ligand dependent signalling of hN2, without affecting ligand independent signalling.

## 2 Results

To analyse the effect of the chosen six mutations (F1565S, L1566P, L1573P, V1623D, I1627N and A1647P) on the mechanical stability of the NRR, relevant to a force-induced Notch activation mechanism, a combined experimental (atomic force microscopy; AFM) and computational (all-atom MD simulation) approach was taken to forcibly unfold the mutated hN2-NRR proteins. The video output representing results from each MD simulation can be found in the Supplemental Data (Movie S1–S7). Analysis of the data highlights three regions of the NRR structure to be particularly affected upon mechanical unfolding following mutation. These observations are discussed in the following sections.

### 2.1 A reduced force is observed for crucial LNRA:B & LNRB unfolding events in half the mutated constructs

**Fig. 2:**
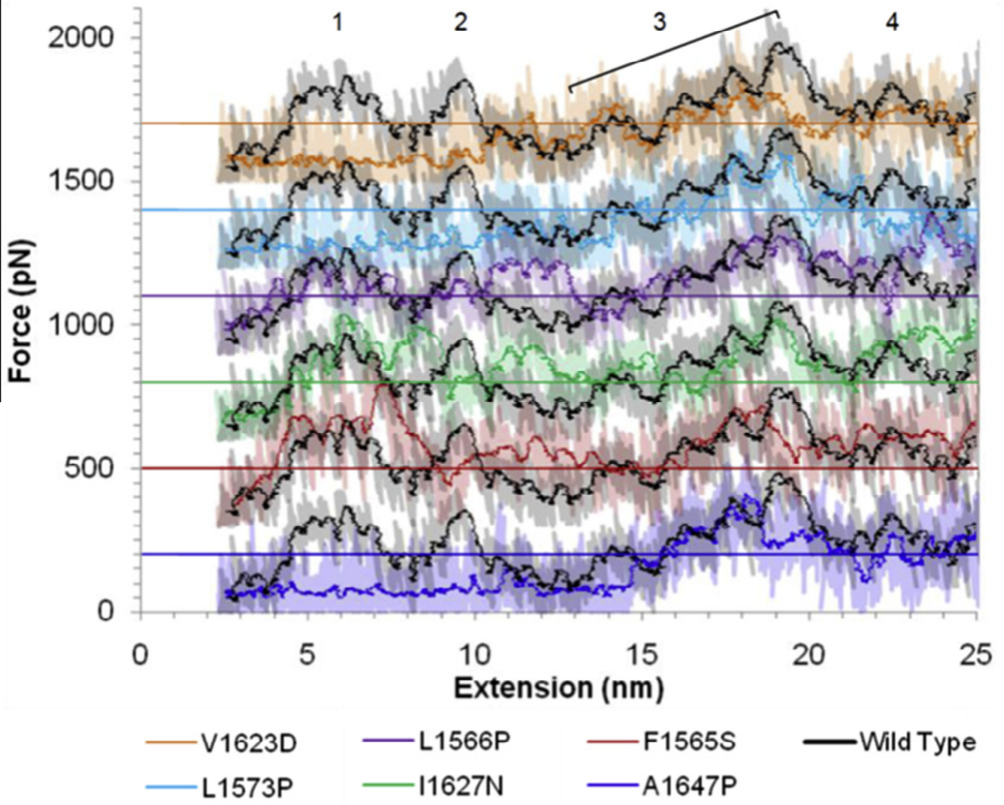
Comparison of the simulated forced unfolding of the hN2-NRR mutated constructs with wild type. Force-extension outputs from the molecular dynamics simulations are shown as follows: F1565S (red), L1566P (purple), L1573P (light blue), V1623D (orange), I1627N (green), A1647P (dark blue), wild type (black). Each graph is staggered by 300 pN with a horizontal line dissecting the plots at 200 pN. Raw data shown (semi-transparent) in addition to a running average (period: 50). Data generated from Gromacs 4.5.3. Peaks in the force-extension outputs are numbered [1–4] according to structural transitions identified from simulation video outputs (Movies S1–S7) and described in the main text and previously (18).

The first key region to be affected by three out of the six mutations investigated is the LNRA:B linker region/LNRB. Force-extension profiles from the MD simulations of mutated constructs compared to the wild-type construct are shown in Fig. 2. NRR-WT shows four distinctive peaks (numbered) corresponding to the unfolding of the LNRA:B linker [1] and LNRB [2], the removal of the β5 strand from the surrounding HD domain [3] and unfolding of the α3 helix and some LNRC unfolding [4], as previously described in detail [18]. A1647P, L1573P and V1623D demonstrate clear loss of force required for the unfolding of the LNRA:B linker and LNRB (peaks 1 and 2, Fig. 2). Crucially, removing the LNRA:B linker and LNRB from their contacts with the HD domain has previously been shown to be sufficient to alter the auto inhibited conformation of the NRR such that the S2 site, within the β5 strand, is accessible to metalloprotease allowing signal activation [18,21]. Thus, these observations indicate that these three mutations within the core region of stability cause destabilisation of regions distant to the mutation site, removing the mechanical resistance to unravelling of this key autoinhibitory region.

**Fig. 3:**
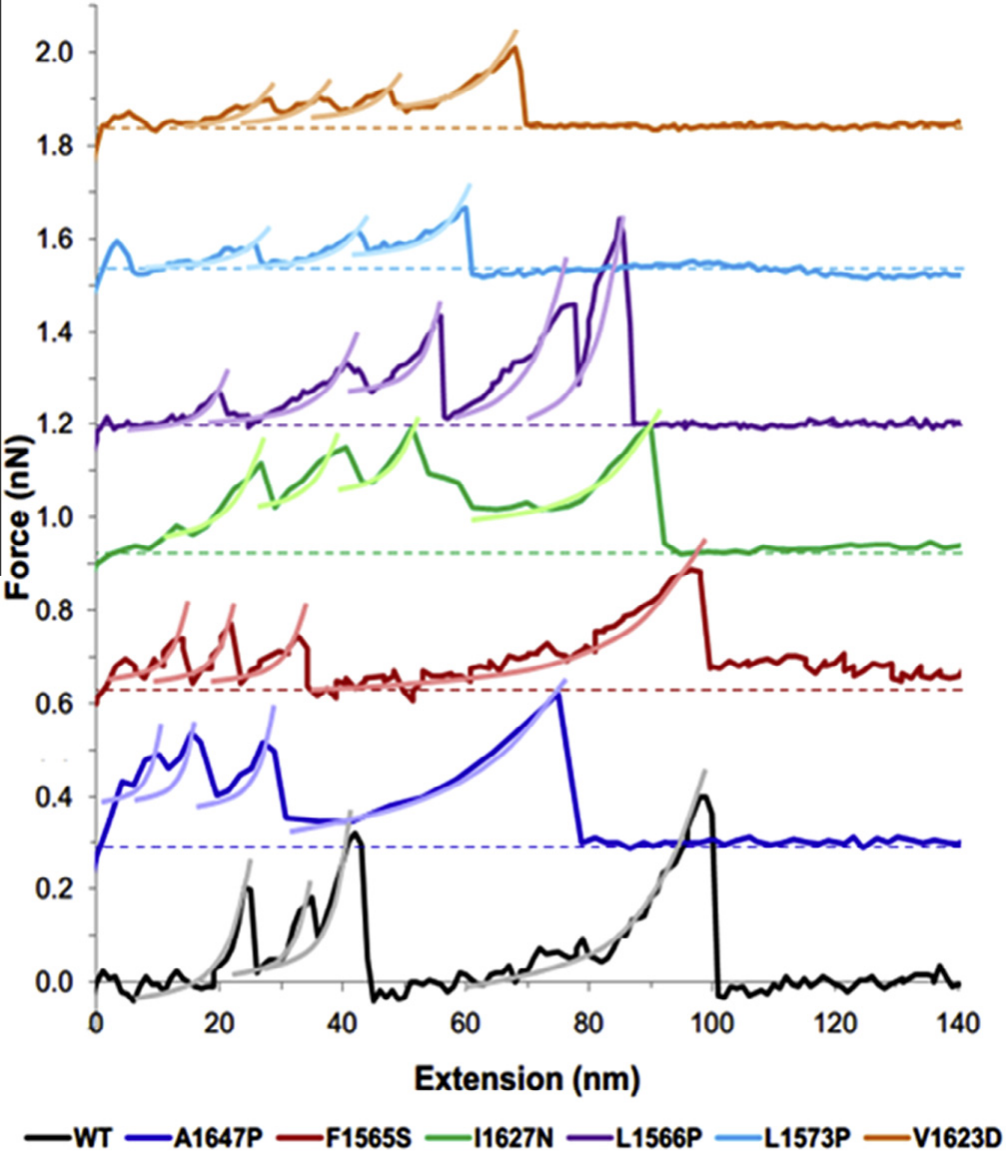
Example raw data curves for each variant construct with the wild type for reference. Each curve is offset by 0.3 nN relative to the previous, with a dashed line highlighting the baseline for each hN2-NRR construct. Worm-like-chain analysis is added to curves as lighter lines.

**Fig. 4:**
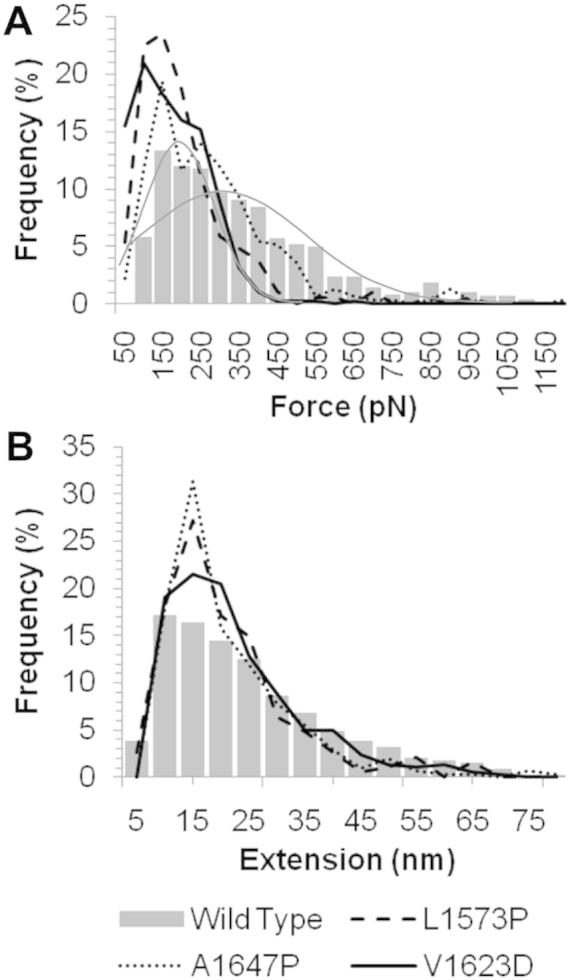
Comparison of the unfolding forces and extensions when wild type and variant hN2-NRR constructs are exposed to AFM unfolding. Frequency of force (A) and extension (B) events occurring during unfolding features in the wild type construct (bar graph) compared to A1647P, L1573P and V1623D (various lines, as shown in key) highlighting a major reduction in force with little change to the extensions observed. A bimodal distribution of the force frequencies for wild type is highlighted by grey lines.

When these simulation results are compared to those observed within the AFM experiments a similar pattern is observed. Example raw AFM force-extension profiles for mechanical unfolding of the WT and mutated hN2-NRR constructs are shown in Fig. 3. It is clear from these raw profiles that the force required for unfolding of the NRR following A1647P, L1573P and V1623D mutations is reduced. Within the A1647P construct this appears to be confined to the LNR unfolding force (first cluster of 3 peaks, Fig. 3), whilst the L1573P and V1623D mutations cause a reduction in the force required for unfolding across the whole construct compared to the WT profile. These data confirm trends observed within the MD simulations, suggesting a destabilisation within these constructs leading to lower forces required for unfolding the LNRA and B regions. Furthermore, analysis of collected sets of such forced unfolding data gained through AFM experiments shows the same three mutations (A1647P, L1573P and V1623D) reduce the force required for unfolding of the NRR compared to the wild type (Fig. 4). Whereas, introducing mutations I1627N and F1565S showed little change (Fig. S1). Unfolding of WT hN2-NRR shows a bimodal distribution (median = 179 and 373 pN). The initial peak in the frequency of the force data has previously been identified as corresponding to the unfolding of LNR modules A and B, whereas the broader distributed peak, at around 350 pN, was identified as HD domain unfolding, possibly combined with LNRC (the broad distribution attributed to non-specific interactions between Lys side chains and the AFM slide during the attachment process [18]). L1573P and V1623D show the greatest reductions in force, lowering the mean force of unfolding from 341.8 to 177.1 pN (L1573P) and 147.4 pN (V1623D). A1647P shows an increase in the number of unfolding events at the lower force of 150 pN as well as a slight reduction in the number of unfolding events requiring higher forces (mean force of unfolding 247.3 pN). These changes in the force required for unfolding between the WT and mutated constructs were all found to be highly statistically significant (*P* < 0.001). This further confirms results observed within the MD simulations indicating a reduction in the force required for unfolding resulting from mutating residues A1647, L1563 and V1623. Furthermore, data suggests this reduction in force is related to the unfolding of the LNR domains, and from the MD simulations can be more specifically located to the removal of the inhibitory plug formed by residues in the LNRA:B linker region and LNRB, key events in removing the auto inhibition and allowing for cleavage at the S2 site. Simulations of the other three mutations (F1565S, L1566P and I1627N) show some reduction in force required for the LNRB unfolding, whilst maintaining the force required for LNRA:B linker removal (Fig. 2). This could also suggest a mechanism by which these mutations could affect the forced unfolding of the NRR, though the effects are less clear in these mutants. Furthermore, AFM for these mutated constructs shows much smaller reductions in the force required for unfolding (Figs. 7 and S1).

### 2.2 Marked destabilisation of the α3-helical region is observed during unfolding of all mutated constructs

**Fig. 5:**
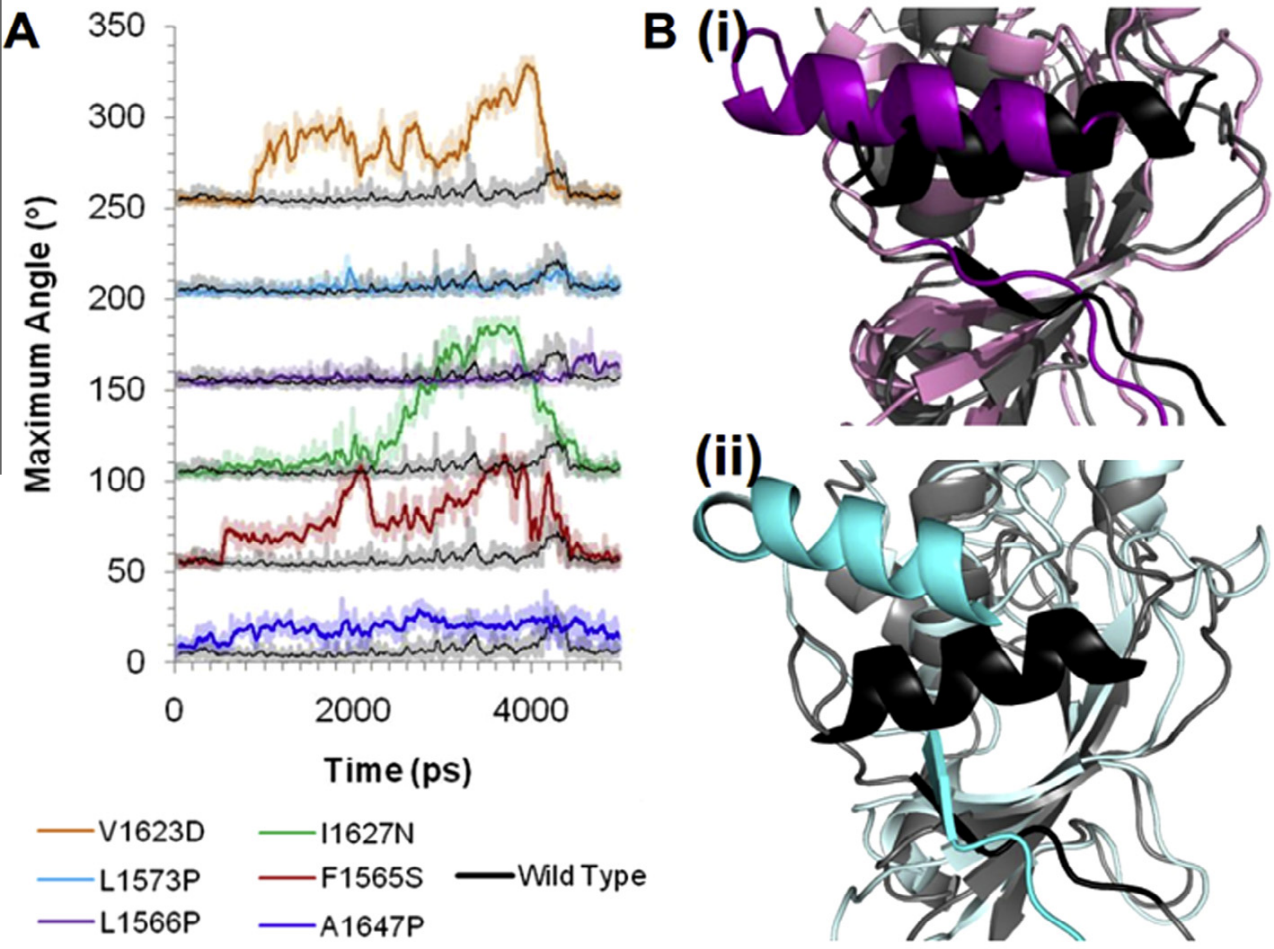
Changes in the angle and position of the α3-helix within variant constructs compared to the wild type during the unfolding simulation. (A) Comparison of the maximum angle across the α3-helix over the course of the pull simulation for each variant and the wild type construct (black). Each comparison is staggered by 50°. Data were generated using the Bendix plugin for VMD [46] from a simulation trajectory gained from Gromacs 4.5.3. (B) Comparison of (i) the wild type and L1566P structures at time 2000 ps; (ii) the wild type and L1573P structures at time 2000 ps. Data generated from Gromacs 4.5.3, images created in PyMol 1.3.

In addition to the observed changes in the LNRA:B linker unfolding we identify significant differences in the structure of the α3-helix of the HD domain during simulated forced unfolding of mutated hN2-NRR constructs compared to the WT (Fig. 5). To analyse the degree of bending occurring across the α3-helix during the simulations the maximum angle across the helix (where a straight helix would have an angle of 0°) was measured for all constructs (Fig. 5A). The WT construct shows minimal bending of the α3-helix during unfolding, with a relatively low angle and minimal changes throughout the simulation (Fig. 5A). In contrast, F1565S, I1627N and V1623D, show large increases in the angle across the α3-helix beginning relatively early in the unfolding process (around 800 ps, corresponding to the time at which the LNRA:B linker has been removed from its contacts with the S2 cleavage site). From structural analysis of these constructs the α3-helix can be observed either partially (F1565S and V1623D) or fully (I1627N) unfolding whilst the α3-helix within the WT NRR remains intact (Fig. S2B–D). This unfolding event occurs before unfolding of the HD domain and appears to be the result of the LNRB domain being pulled from the structure (Movie S1, S3, S4 and S7). A1647P shows a smaller general increase in the angle across the α3-helix throughout the simulation, compared to the other mutations discussed. Furthermore, the α3-helix of A1647P is observed unfolding in a similar manner to the WT construct (Fig. S2A and Movie S1 and S2). The difference in angle observed for A1647P is likely the result of the proline residue introduced within the α3-helix in this construct. Interestingly, in addition to the introduced kink in the α3-helix, this mutation also causes the helix to rotate upwards towards LNRB, which could cause wider destabilising effects. Finally, L1566P and L1573P show little change to the angle across the α3-helix during the unfolding simulation; however, structural analysis shows a positional shift of this α3-helix, in both constructs (Fig. 5B). The observed shift brings the α3-helix away from the β5-strand, which houses the S2 site, allowing greater exposure of this cleavage site. Overall, the α3-helix would thus appear to either be structurally destabilised or positionally changed during mechanical unfolding following mutation at all six HD domain sites, potentially resulting in greater access to the S2 cleavage site.

### 2.3 Two mutations affect the association of LNRC with the HD domain

**Fig. 6:**
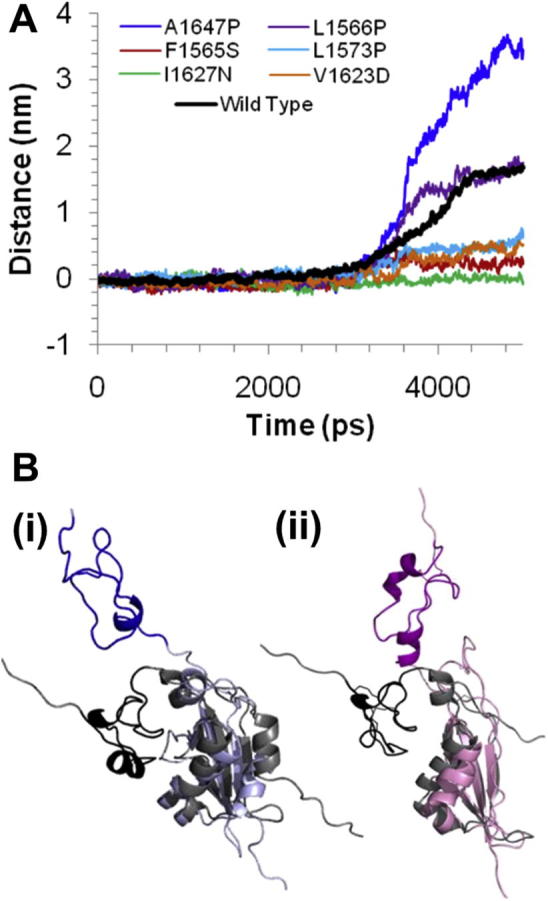
Observed differences in unfolding of LNRC for hN2-NRR A1647P and L1566P. (A) Comparison of distances between the LNRC and HD domain between wild type and variant NRR constructs. (B) Comparison of the wild type and (i) A1647P NRR structure at time 4000 ps and (ii) L1566P NRR structures at time 5000 ps.

The final change observed during simulated unfolding following HD domain mutation is within the LNR:HD domain interaction. During simulated forced unfolding, the WT hN2-NRR shows a steady increase in distance between the LNRC and HD domain (using the starting centre-of-mass for each domain) from 2800 ps until 4400 ps, where it plateaus at 1.5 nm (Fig. 6A), however, changes observed within the structure are minimal (Fig. S3). The same data recorded for the six mutated protein constructs draws attention to two constructs in particular that have an apparent decreased stability for the LNRC:HD association (A1647P and L1566P) with increases observed in the LNRC:HD domain distances. Upon structure analysis of the LNRC:HD domain contacts within A1647P and L1566P large differences are observed relative to WT (Fig. 6B). The loss of stability of the LNRC:HD domain contact results in unfolding of LNRC, an event not observed within simulations of equivalent duration in all other constructs, including WT (Movies S1–S7). Interestingly, calculated distances between the HD and LNRC domain of L1566P (Fig. 6A) results in a final distance equivalent to that of the WT construct, despite the observable differences in the structures (Fig. 6B(ii)). This appears to be the result of a shift in part of the HD domain structure causing a shift in the centre-of-mass. As the LNRC of L1566P unravels and dissociates from the HD domain, part of the HD domain also raises up away from the HD domain structure, altering the position of the centre-of-mass in this domain and resulting in the reduced final distance measurement. This paradox illustrates the difficulty in using distances between two centres-of-mass within the protein structure, as regions respond to force differently. F1565S, I1627N, L1573P and V1623D exhibit a decrease in the distance between the two regions over the unfolding time course which corresponds to minimal structural changes similar to WT (Suppl. Movies S1, S3, S6 and S7). The differences in distance observed are likely due to changes in the centre of mass within the HD domain as other unfolding events occur (e.g. β5 strand removal from the main HD domain structure).

**Fig. 7:**
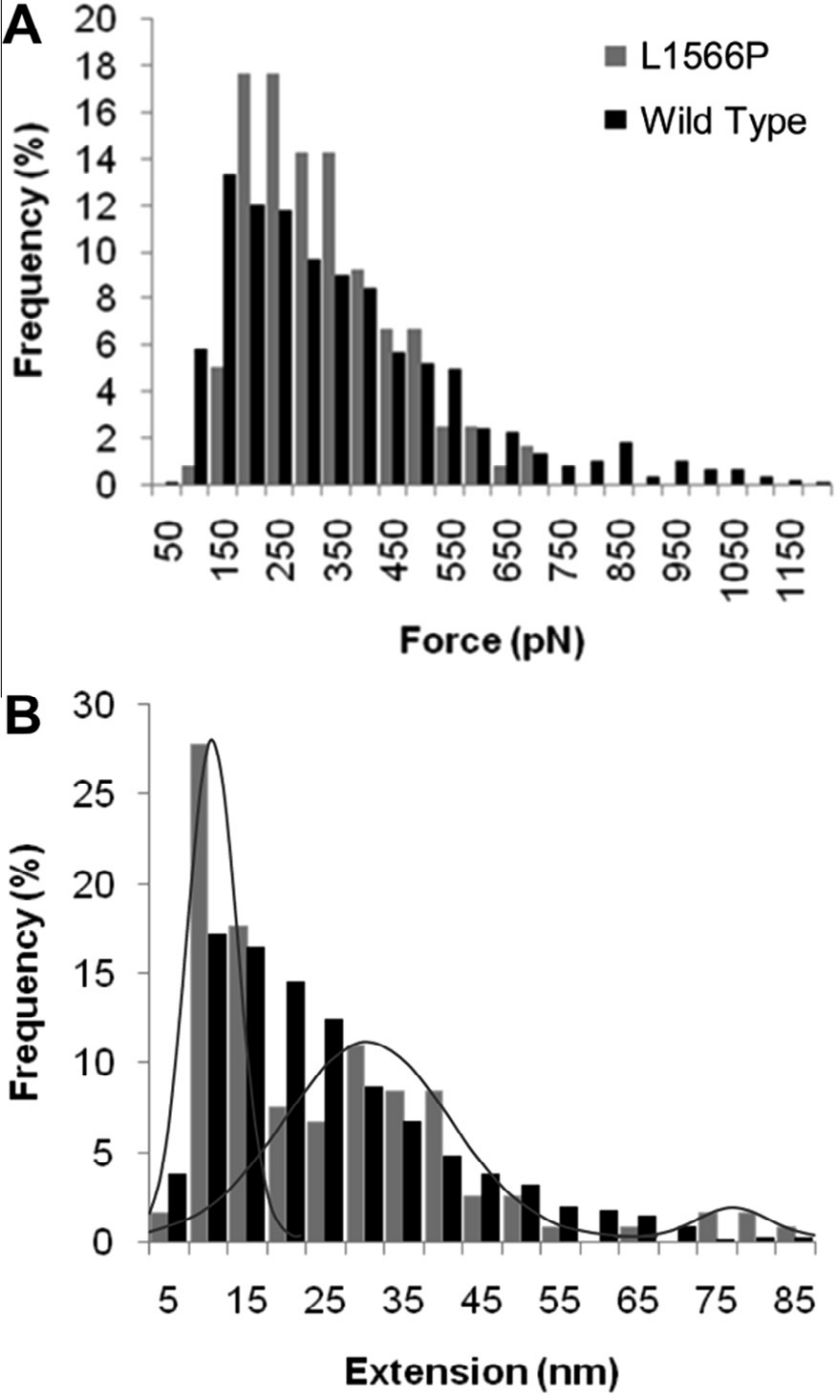
AFM force and extension data for L1566P compared with the wild type construct. Comparison of the frequency of force (A) and extension (B) events observed during unfolding features of the wild type and L1566P constructs showing a change in distribution of the extension profile, resulting in three distinct peaks (highlighted by grey lines).

Examination of the AFM data obtained for L1566P shows a reduction in the number of unfolding events requiring a force higher than 500 pN (Fig. 7A), in agreement with a destabilisation of structure. In addition, observed differences in extension lengths could also correlate to the reduced LNRC:HD stability observed in the MD simulation (Fig. 7B). In the WT construct, the initial peak in frequency at 10 nm corresponds to LNRA and LNRB unfolding events, with a slow reduction in frequency at larger extensions causing a trailing tail. This tail has previously been attributed to non-specific interactions between lysine residues present in the HD domain and the functionalised AFM slide [18]. We have also previously suggested that the larger extensions correspond to overlapping unfolding of the HD domain and LNRC. In L1566P it is notable that the frequencies are now more centred around three peaks and not distributed as broadly to give the trailing tail (Fig. 7B, highlighted by grey lines). The initial LNR peak at 10 nm is still present, followed by a second distinct peak at around 30 nm. A final peak is also observed around 80 nm. Taking these data together with the MD simulations, it is possible that in the WT construct, the trailing tail does indeed result from unfolding of the LNRC together with part of the HD domain, and that when a mutation disrupts the interactions between the HD domain and LNRC, this overlap in unfolding is affected. Hence, the 30 nm ‘peak’ in extension frequency may correlate to HD domain unfolding. The increase in the number of unfolding events at higher (80 nm) extensions is harder to explain. It is possible that the reduced interaction between LNRC and the HD domain results in loss of features in the AFM unfolding profile (and loss of trailing tail) since the force required to disrupt it is too low to be detected by AFM. The extension increase for this event will add to the extension observed for subsequent unfolding events, thereby increasing the total extension observed for these events. It should be noted, however, that the final peak occurs at very low frequency, to a degree that statistical analysis does not support its significance, relative to the WT profile; therefore all conclusions are purely speculative.

Although a decrease in the force required for unfolding A1647P relative to WT is observed within the AFM unfolding data (Fig. 4A), little change is seen for the extension profile (Fig. 4B), which contrasts to data for L1566P. As both of these variants show an increase in the level of LNRC unfolding in simulations, similar AFM extension profiles were expected. However, it is possible that mutation of residue 1647 has a more indirect effect on the stability of LNRC which is not sufficient to affect the LNRC:HD domain interaction and separate the overlapping unfolding of LNRC and the HD domain to detectable levels in AFM experiments (residue L1566 is located directly at the LNRC:HD domain interface, whilst A1647 is remote to it, in the α3-helix). The large difference in the stability of the LNRC:HD domain association observed for A1647P does, however, indicate significant destabilisation of the HD domain, with structural changes that impact on the unfolding pathway.

## 3 Discussion

An increasing quantity of evidence supports a force-driven mode of activation of the Notch signalling pathway, whereby exposure of the critical S2 site in the Notch NRR occurs upon mechanical unfolding of the protein during transendocytosis of the Notch extracellular domain into the ligand presenting cell. Having directly examined the mechanical unfolding and cleavage of the hN2-NRR in proof-of-feasibility of this proposed activation mechanism [18], we now increase insight into the mechanical properties of the NRR by combining experimental and computational unfolding studies with mutagenesis. Mutations were made to hN2-NRR at residue positions previously shown to cause a gain-of-phenotype in hN1 activation [6,7]. The equivalent mutations in hN1 affect the stability of the NRR, specifically the HD domain, as demonstrated by more ready dissociation of S1-cleaved hN1-NRR variants into two subunits, HD-N and HD-C [28]. Given the location of the majority of the mutated residues in the core α1 helix or β4 strand (Fig. 1), disruption of HD-N and HD-C association on mutation is not surprising. Interestingly, in biochemical studies of three of these hN2 equivalent hN1-NRR mutants the ligand independent activation was no greater than basal N2 WT levels [40]. This suggests interesting differences between hN1 and hN2 with regards to chemical stability. However, as ligand induced activation of the Notch pathway could strongly depend on the NRR withstanding mechanical force, it is important to study the effect of such mutations on the mechanical stability of the NRR and relate this back to Notch activation and S2 site exposure/cleavage. On examination of our forced unfolding data, all mutations decrease the mechanical stability of the hN2-NRR. Simulations in particular point to three key regions of structure or tertiary interactions that are more markedly affected in the mutated constructs relative to WT NRR during forced unfolding (Fig. S4 and Table S1). The mutations are thus categorised and discussed in relation to these key regions in turn, which are: the LNRA:B linker/LNRB region, the HD domain α3 helix and LNRC.

With respect to the LNRA:B linker/LNRB, half of the hN2-NRR mutations made (A1647P, L1573P and V1623D) caused a large and significant reduction in the force required to ‘unplug’ this linker and the LNRB away from the HD domain. In AFM experiments, the force required for NRR unfolding is dramatically reduced compared to WT. In MD simulations, the force barrier attributable to LNRA:B linker removal, identified in previous analyses with WT hN2-NRR as the first significant peak in the force profile, is no longer detectable in the three variants. Furthermore, a significant reduction is observed in the second unfolding peak, corresponding to the removal of the LNRB from its contacts with the HD domain (as described previously, 18). Our previous simulation study highlighted removal of the LNRA:B linker as the minimal structural change required to allow metalloprotease cleavage at the S2 site [18]; whilst biochemical studies showed additional removal of the LNRB was absolutely required for S2 cleavage [21]. Together, this data suggests that all three variants could cause increased Notch signal activation as a greatly reduced unfolding force is observed *in vitro*, which likely corresponds to the reduced forced required for both LNRA:B linker and LNRB removal as observed *in silico*. Whilst *in vitro* evidence indicates that the unwrapping of LNRA and LNRB away from the HD domain is necessary and sufficient for S2 site exposure and cleavage by metalloprotease [18], the disruption of other parts of the NRR structure on experiencing a mechanical force could, of course, also affect S2 site exposure. Indeed, MD simulations on the WT hN1-NRR [20] led the authors to propose that unfolding of LNRC and the α3 helix within the HD domain were possible pre-requisites for optimal exposure of S2. The α3 helix packs on to the β5 strand housing the S2 site and, as such, it is a clear candidate for increased accessibility to the S2 site upon structural perturbation. A striking observation for all mutations in our study is a decreased mechanical stability of the α3-helix of the HD domain within these mutated constructs. The majority of the mutations cause an increased degree of unfolding within this helix, relative to WT, within the same time-course, whilst L1566P and L1573P resulted in a greater extent of movement in this region causing it to shift away from the S2 cleavage site. The large differences observed within this region in all six of the mutations studied here suggest an important role for the α3-helix in the structural integrity of the NRR.

Two hN2-NRR variants reduce the mechanical stability of LNRC, which is observed to dissociate from the HD domain and unfold in MD simulations (an event not actually observed within the wild type construct during the time-course of simulated forced unfolding). The L1566P mutation is located within the α1-helix adjacent to the Arg1567 residue, shown previously to interact with residue Asp1506 within the LNRC to form a salt bridge [21]. The Leu to Pro substitution at position 1566 could very well interfere with salt-bridge formation, allowing LNRC to more readily dissociate and move away from the HD domain as force is maintained. AFM unfolding data concurs with this proposition, showing a loss of unfolding events previously hypothesised to correspond to combined LNRC and HD domain unfolding [18] and replacing them with two distinct unfolding events, one of which occurs at greater extensions. Whilst the salt bridge is also observed in the hN1-NRR [22], previous mechanical unfolding data on hN1-NRR unfolding, both simulated [20] and experimental [41], reveals a different unfolding pathway to hN2-NRR, whereby in hN1 the unfolding of the three LNRs is clearly sequential, followed by HD domain unfolding. Furthermore, as mentioned earlier the L1566P mutation does not result in a gain-of-phenotype in the hN2 ligand independent pathway [40]. These fundamental differences are striking; however, there is a clear role for the salt bridge in increasing the mechanical stability of the Notch NRRs. Destabilisation of the hN2 salt bridge would, therefore, result in increased NRR unfolding. In hN2, but not in hN1, the LNRC:HD interaction is further stabilised via a zinc-ion and we have previously suggested that this increased stability contributes to the differences in the unfolding pathway observed for hN2-NRR versus hN1-NRR. *In vitro* studies looking at the functional effect of the coordinated ions conclude that whilst removal of the calcium and zinc ions with EDTA treatment dramatically affects the mechanical unfolding of the hN2-NRR [18], it does not allow for ligand independent activation in the absence of force as observed for hN1 [40]. We argue that the effect of the L1566P mutation on simulated and experimental unfolding data, observed here, again points to the LNRC:HD interaction as having a key bearing on the number and order of unfolding events in the unfolding pathway of NRR constructs. Such differences in the response to mechanical force of the hN1 and hN2 NRRs, as highlighted by the influence of the salt-bridge and metal ions, could have functional implications with respect to the mechanism and regulation of their activation [42,43].

Interestingly, A1647P is located within the α3-helix of the HD domain yet affects the LNRC:HD domain interaction, the site of which is some 24 Å away. AFM data of A1647P shows a large reduction in the force observed for the unfolding of the NRR, compared to WT, and the LNRC is observed to move away from the HD domain in simulated unfolding of A1647P (Fig. 6). The mutation thus appears to cause large destabilisation within the NRR. The AFM data are quite different to those observed for L1566P, indicating that the explanation or cause of this destabilisation is different. Indeed, A1647P shows both loss of any force required to remove the LNRA:B linker and an altered positioning of the α3 helix during the forced unfolding process, relative to WT. Both of these processes can be explained by location of A1647 in the α3 helix itself, close to the interface with the LNRA:B linker. It is possible that destabilisation of the association of LNRA and LNRB with the α3 helix could lead to easier removal of LNRC from the HD domain as force is applied, pointing to a pivotal role for the α3 helix in mechanical stability and regulated exposure of the S2 site.

Note that the hN2-NRR proteins in this study have an intact S1-site and are not treated with furin. The importance of furin cleavage still requires full understanding [44]. S1-resistant forms of hN1 are reduced in signalling competency, whereas S1-resistant hN2 are not [45], indicating that its importance might vary between Notch receptors. There currently exists very little in the way of evidence for a role of the furin cleavage event in the activation of the Notch signal. Expression of the L1566P, V1623D and I1627N N2 receptors in U2OS cells showed a reduction in S1 cleavage efficiency compared to WT N2, resulting in lower expression at the cell surface [40]. In addition, the hydrogen-exchange mass spectrometry studies referred to earlier could detect no difference between hN1-NRR proteins that were or were not S1-cleaved, with respect to how structures switch from auto inhibited conformation to a mimic of an activated ‘endstate’ on metal ion removal [27]. Therefore, the S1 cleavage of hN2 could primarily be involved in receptor processing. Certain mutations within hN1 are known to cause dissociation into HD-N and HD-C subunits extremely readily (namely, the equivalent of the L1573P and V1623D constructs) relative to WT [28], which could cause the increased Notch activation observed. Here, with no cleavage at S1, we can only analyse mechanical unfolding and show that mutations reduce the force required for exposure of the S2 site. Our studies cannot establish whether *in vivo* these particular mutations activate signalling primarily *via* decreased mechanical stability or a direct increase in the subunit dissociation rate (perhaps also enhanced by mechanical force), which can expose S2. However, a comparison of the biochemical data published previously and this structural data highlights the potential for different unfolding mechanisms based on the stress they are subjected to. A number of these mutations, despite showing little change in the ligand-independent activation of hN2 [40], reduce forced unfolding dramatically, suggesting different structural elements may be important for mechanical unfolding versus chemical unfolding.

In conclusion, our studies provide insight into key structural elements within the Notch NRR for conferring mechanical stability. The mutations studied all cause destabilisation of one or more of the key regions within hN2-NRR during mechanical unfolding, leading to a more accessible S2 cleavage site. The observations highlight differences between the ligand independent and ligand induced notch signalling pathways and add further weight to a force activation model for Notch signalling. The clear next step is to utilise these single molecule approaches on live cells as recently performed by Gordon et al. [47], to investigate the differences in the forces required for proteolytic cleave of the Notch1 and Notch2 NRR’s.

## 4 Experimental procedures

### 4.1 Protein expression and purification

Recombinant NRR wild type (WT) and variant constructs were produced as GST-fusion proteins with N-terminal poly-lysine (Lys_3_) and C-terminal hexahistidine (His_6_) tags through expression in T7 Express *Escherichia coli* cells (NEB), as previously described [18]. Protein was purified from soluble lysate through incubation with glutathione beads (GE Healthcare), cleaving the GST-tag to release the protein. To gain further purity the protein constructs were denatured and purified further with ion-exchange (Ni^2+^-His; His-Trap™ HP columns) and size exclusion chromatography (S-200 Superdex) (columns from GE Healthcare). Purified protein constructs were refolded in a redox buffer (containing 5 mM Cysteine, 1 mM Cystine and 50 mM CaCl_2_) before structural characterisation (^1^D-^1^H NMR, 2D ^1^H-NOESY NMR, CD, UV Resonance Raman Spectroscopy) was performed to confirm folded protein.

### 4.2 AFM experiments

Gold coated AFM tips were functionalised with Ni^2+^-NTA as previously described [18]. With attachment of the C-terminal end of the HD domain to the AFM tip, the application of force could be directly targeted to the end of the LNRA which is attached to the AFM slide via its poly-Lys tag. In the native situation, a force would be transmitted via the EGF-like repeats, again to the N-terminal side of LNRA. Force experiments were performed on a Nanoscope V controller in PBS buffer at a loading rate of approximately 1 × 10^−7^ N/s (spring constant ∼67 pN/nm; velocity ∼1600 nm/s), with a 2 s surface delay to ensure Nickel-His coordination (based on previous experiments). Buffer conditions retained the necessary metal ions (Ca^2+^ and Zn^2+^) for protein stability. Controls were performed without protein present to determine the level of features occurring due to surface adhesion contacts and to distinguish these from protein unfolding features observed when protein is present. Attachment controls were also performed using competing ions to disrupt Ni^2+^-His interactions at the tip. Statistical significance was determined with the Mann Whitney U Test on each data set compared to that of the WT construct.

### 4.3 Molecular dynamics simulations

Mutated protein constructs were created from the hN2 NRR template (PDB:2OO4) using Swiss Model PDB Viewer version 4.0. MD simulations were performed on WT and mutated hN2 NRR constructs using Gromacs [29] version 4.5.3 with force field gromos96 53A6 parameter set [30]. Short-range interaction cutoff was set to 1.4 nm and long range electrostatics were calculated with the particle mesh Ewald algorithm [31,32]. Dispersion correction was applied to energy and pressure terms to account for truncation of van der Waals terms. Periodic boundary conditions were applied in all directions.

Each protein construct (coordinates) was placed in a 3-dimensional box (dimensions: 10 × 10 × 50 nm) of 100 nM NaCl in simple point charge water [33], including neutralising counterions. Steepest descent energy minimisation was performed followed by a two-step equilibration, with position restraints applied to heavy atoms. Equilibration step one simulated 100 ps under the NVT ensemble (maintaining a constant number of particles, volume and temperature). Temperature was maintained at 310 K (37 °C) by coupling protein and non-protein atoms to separate temperature coupling baths [34]. Equilibration step two simulated 100 ps under the NPT ensemble, maintaining a constant isotropic pressure of 1.0 bar (weak coupling). All position restraints were then removed, except for those on the atoms of the C-terminal residue (Thr1672), which was used as an immobile reference for the pull simulation. For each simulation the atoms of the N-terminal residue (Cys1425) were pulled along the *z*-axis, mimicking the *in vitro* experiments, at a loading rate of ∼1 N/s (spring constant: 1.66 × 10^−9^ N/nm; velocity: 1 × 10^9^ nm/s. These simulations used the Nosé-Hoover thermostat [35,36] and the Parrinello-Rahman barostat [37,38].

## Author contributions

JMA designed the research hypothesis; NLS and JMA designed the experiments, and wrote the paper. NLS performed the experiments and analysed the data.

## Appendix A Supplementary data

Supplementary Movie S1 MD simulated unfolding of hN2-NRR wild type.

Supplementary Movie S2 MD simulated unfolding of hN2-NRR A1647P.

Supplementary Movie S3 MD simulated unfolding of hN2-NRR F1565S.

Supplementary Movie S4 MD simulated unfolding of hN2-NRR I1627N.

Supplementary Movie S5 MD simulated unfolding of hN2-NRR L1566P.

Supplementary Movie S6 MD simulated unfolding of hN2-NRR L1573P.

Supplementary Movie S7 MD simulated unfolding of hN2-NRR V1623D.
