# Supplementary material for: Mutational analysis of the Notch2 negative regulatory region identifies key structural elements for mechanical stability
Source: FEBS Open Bio. 2015 Jul 30;5:625–33. doi: 10.1016/j.fob.2015.07.006 (PMC4537882; doi:10.1016/j.fob.2015.07.006)
Supplement: Supplementary Material — Supplementary Figs. S1–S4 and Table S1. [file mmc8.doc]

**Supplemental Data**

Mutational analysis of the Notch Negative Regulatory Region identifies key structural elements for mechanical stability that are of relevance to S2 protease site exposure.

*Natalie L. Stephenson^*^ and Johanna M. Avis*

Faculty of Life Sciences, Manchester Institute of Biotechnology, University of Manchester, 131 Princess Street, Manchester M1 7DN, United Kingdom.

^*^ **To whom correspondence should be addressed:** Natalie L Stephenson, Cancer Research UK Manchester Institute, Wilmslow Road, Manchester, M20 4BX, United Kingdom. Tel.: +44 (161) 4463039; Email: natalie.stephenson@manchester.ac.uk

**Running Title: Notch NRR mutations shed light on S2 site exposure**

**Abbreviations:** NRR, Notch Regulatory Region; TACE, TNF-alpha-converting enzyme; hN1, human Notch-1 receptor; hN2, human Notch-2 receptor; HD, heterodimerization domain; LNR, Lin12-Notch Repeats.

**Key words:** Notch, Negative Regulatory Region, S2 cleavage site, Atomic Force Microscopy, Molecular Dynamics Simulations.

**Fig. S1.** Comparison of the unfolding forces and extensions when wild type and
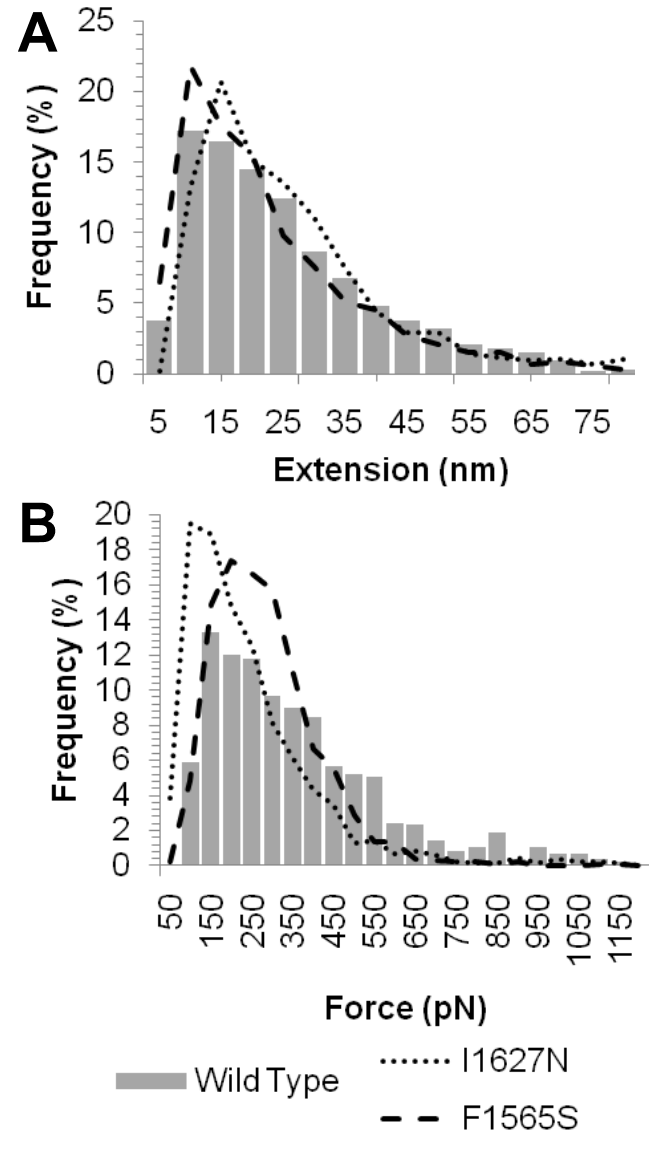
mutant NRR constructs are exposed to AFM unfolding. Frequency of force **(A)** and extension **(B)** events occurring during unfolding features in the wild type construct (bar graph) compared to mutants F1565S and I127N highlighting a significant reduction in force with little change to the extensions observed. AFM data for all other mutant hN2-NRR constructs are provided in the main manuscript.


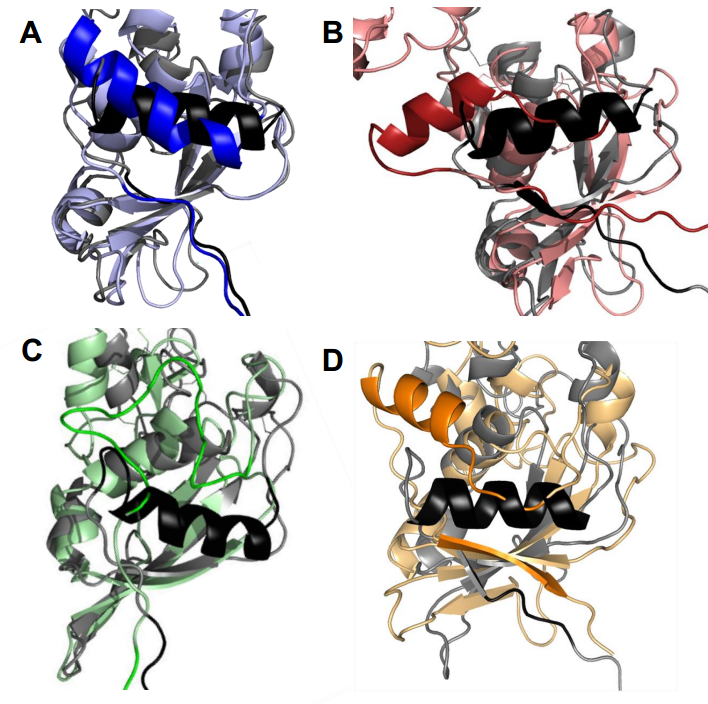


**Fig. S2.** Comparison of the α3-helix position and unfolding of wild type and mutant constructs. Position of the α3-helix during mutant unfolding (at 2000 ps) compared to the wild type construct (black) for mutants (A) A1647P, (B) F1565S, (C) I1627N and (D) V1623D. Data generated in Gromacs 4.5.3, images created in PyMol 1.3.

**
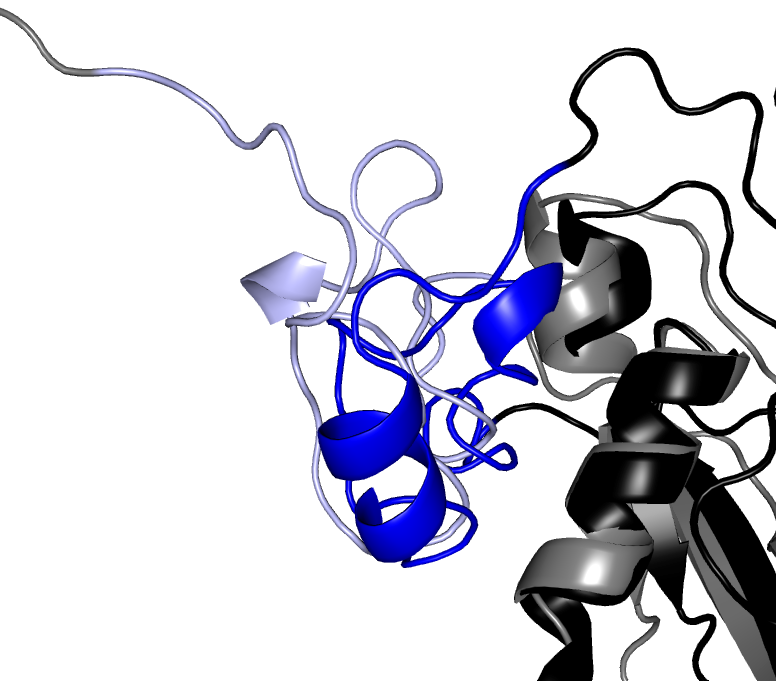
**

**Fig. S3.** Comparison of the structures of wild type hN2-LNRC at the start (t = 0 ps, dark colour) and end (t = 5000 ps, light colour) of the MD simulations. The LNRC (blue) within the wild type construct changes very little over the course of the simulation. Images created in PyMol 1.3.


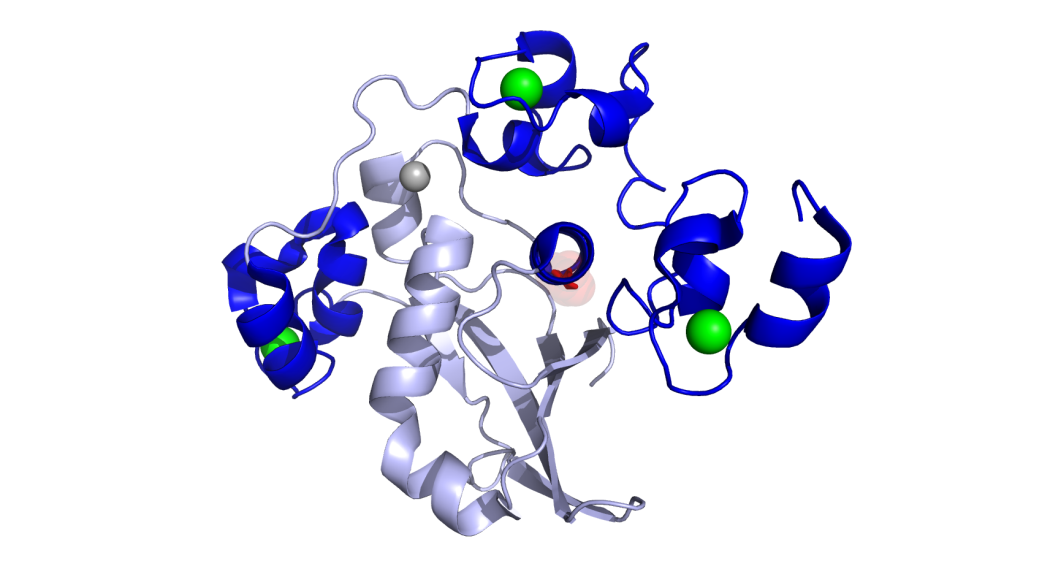

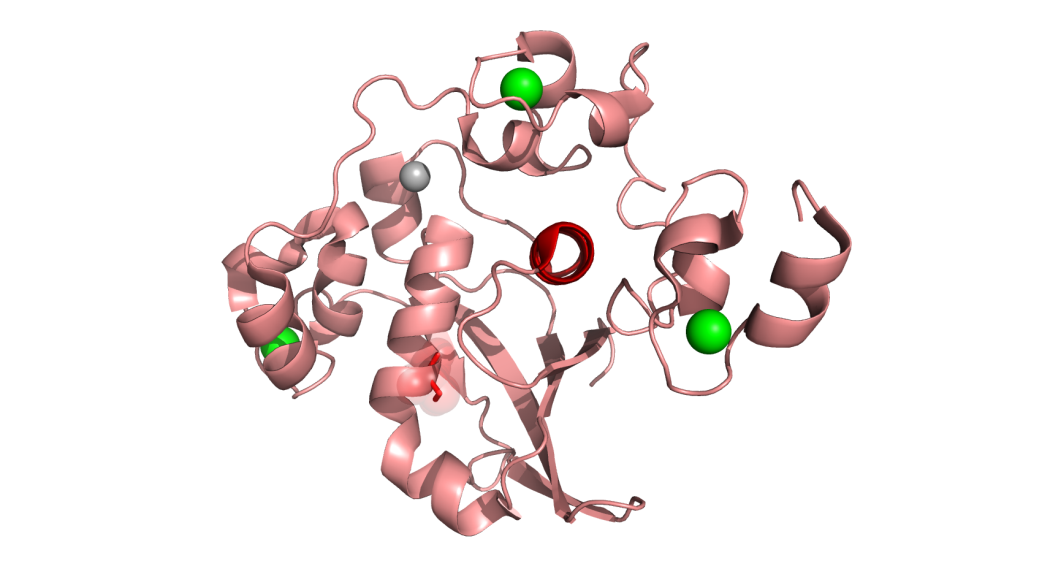

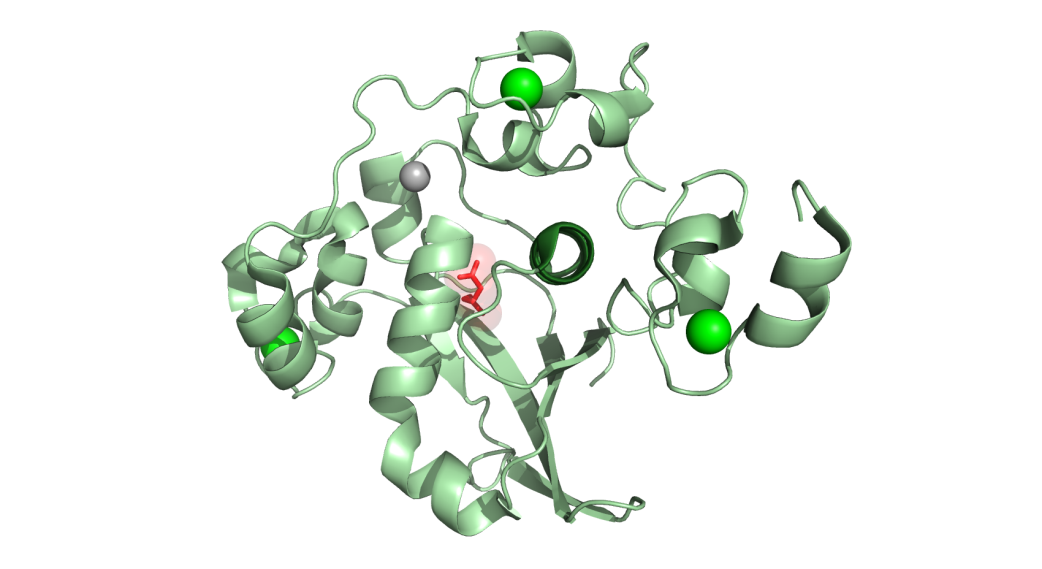

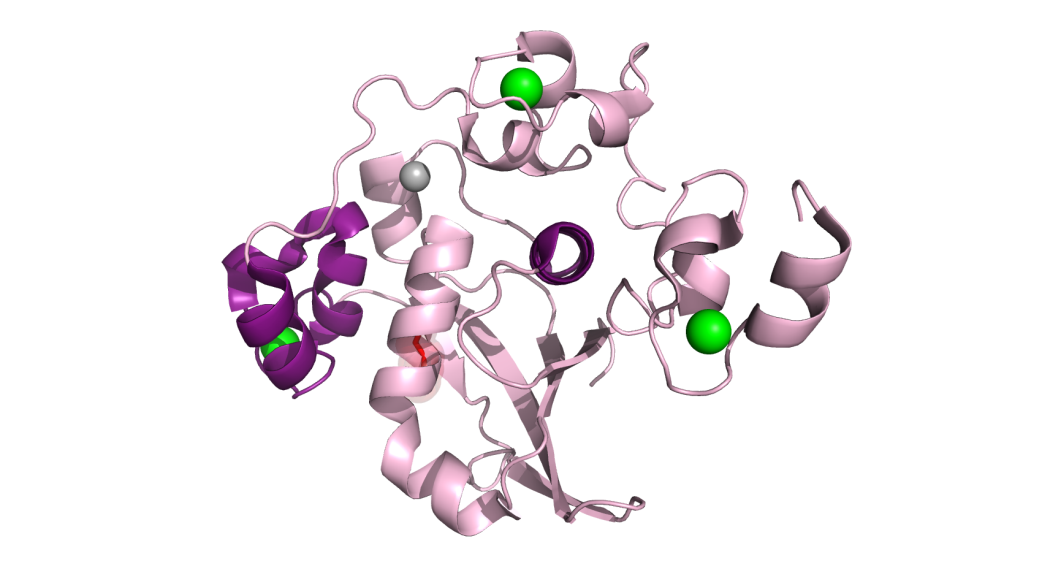

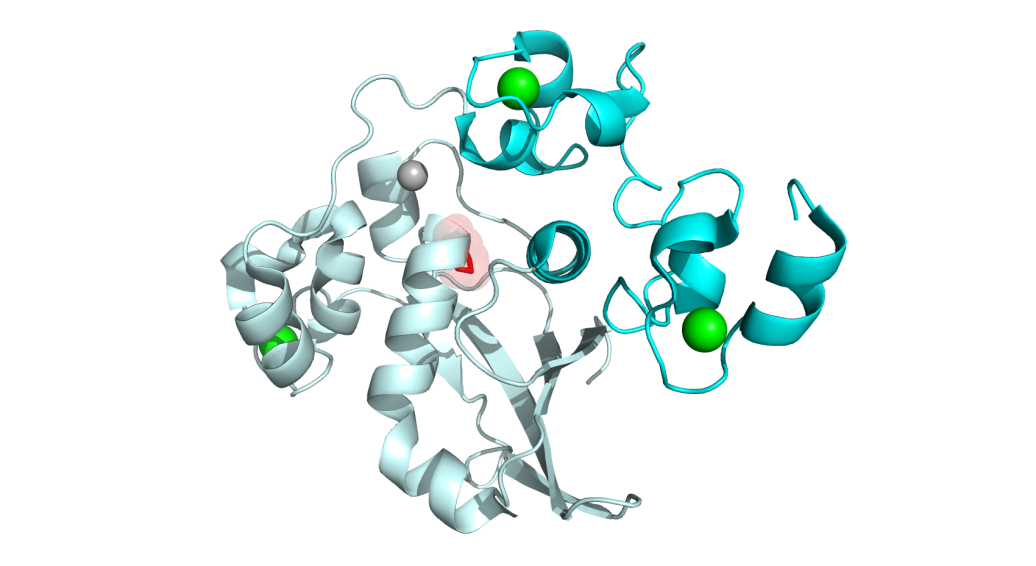

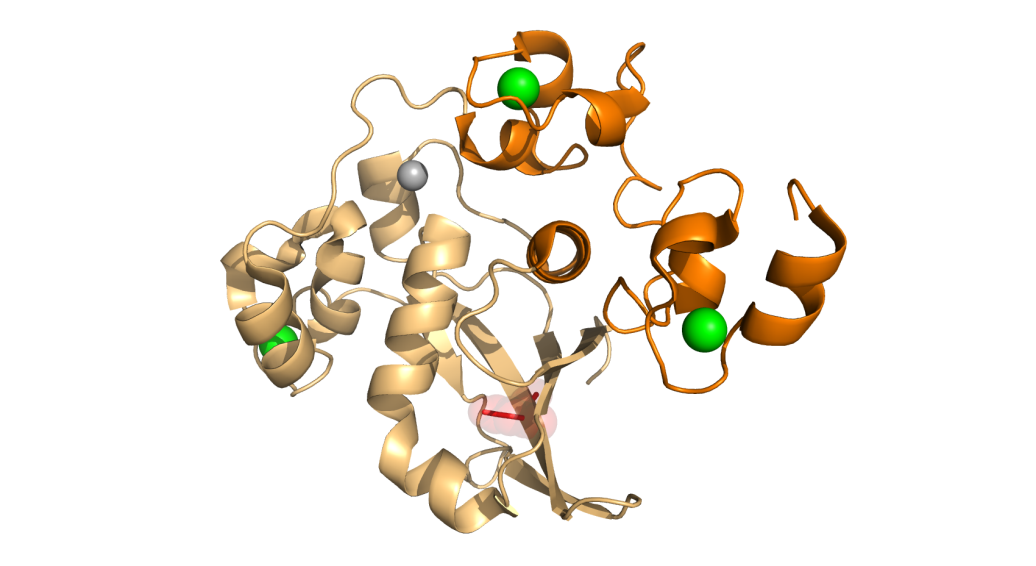


**A**

**B**

**C**

**D**

**E**

**F**

Fig. S4. Comparison of the mutation site (red) and the regions of the protein showing altered structural changes (darker colour) during the forced unfolding simulations when compared to the WT for each of the mutations analysed. (A) A1647P, (B) F1565S, (C) I1627N, (D) L1566P, (E) L1573P and (F) V1623D. Images created in Pymol 1.3

**Table S1. A comparison of structural data presented within this paper and previous studies on the same/equivalent mutations within N2 and N1.**

| **Mutation** | **Simulations** | | | | **AFM** | | **Biochemically** | |
| --- | --- | --- | --- | --- | --- | --- | --- | --- |
|  | **LNRA:B** | **LNRB** | **LNRC** | **α3-helix** | **Force** | **Extension** | **Notch 2** ***^a^*** | **Notch 1** ***^b^*** |
| F1565S | - | - | - | Shift and partial unfold | Slightly lower | - | - | 2 M |
| L1566P | - | - | Unfolds | Shifted | Slightly lower | - | No change | 2.5 M |
| L1573P | Lower force required | Lower force required | - | Shifted | Lower | - | - | 0 M |
| V1623D | Lower force required | Lower force required | - | Shift and partial unfold | Lower | - | No change | 0 M |
| I1627N | - | - | - | Fully unfolds | Slightly lower | - | No change | 1.5 M |
| A1647P | Lower force required | Lower force required | Unfolds | Kinked and rotation/shift | Lower | - | - | 3M |
| *^a^* Data from luciferase reporter assays in U2OS cells transfected with Notch 2 WT and mutant constructs when exposed to EDTA (40). Change in luciferase signal is stated.  *^b^* Data from Notch 1 mutant NRR domains expressed and secreted from U20S cells as cleaved heterodimers, compared with WT Notch 1. Urea concentration under which heterodimer dissociation is observed is stated (28). | | | | | | | | |
